# Primary Squamous Cell Carcinoma of the Urinary Bladder Presenting as Peritoneal Carcinomatosis

**DOI:** 10.1155/2010/179250

**Published:** 2010-06-27

**Authors:** Himisha Beltran, Brian D. Robinson, Scott T. Tagawa

**Affiliations:** Weill Cornell Medical College, USA

## Abstract

We report an unusual case of a 78-year-old Caucasian female, who presented with peritoneal carcinomatosis and hypercalcemia, and was found to have a rapidly progressive primary squamous cell carcinoma of the urinary bladder. Squamous cell bladder carcinoma is a rare malignancy in the United States, accounting for just 1–3% of bladder tumors. Interestingly our patient lacked the established risk factors, including exposure to the parasite *Schistosoma haematobium*, recurrent urinary tract infections, bladder calculi, radiation exposure, chronic indwelling catheter, neurogenic bladder, or tobacco abuse. Although hypercalcemia has been rarely described, an initial presentation of peritioneal carcinomatosis has not been previously reported.

## 1. Case Report

**Figure 1:**
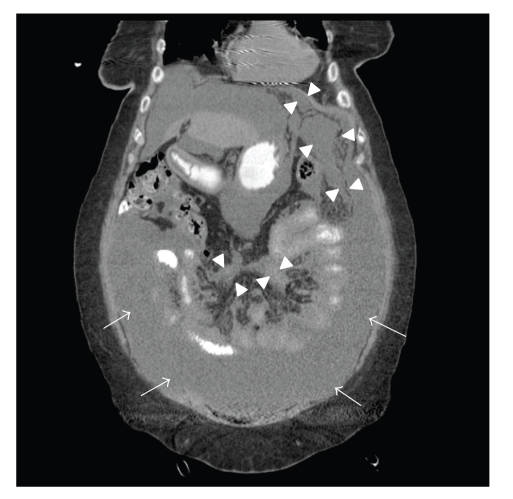


A 78-year-old Caucasian woman presented with a subacute onset of diffuse abdominal pain, associated with progressive abdominal distention, nausea, and anorexia. Her initial labs were notable for hyponatremia (Sodium level of 127 mEq/L), and hypercalcemia (corrected calcium level of 14 mg/dL), and acute prerenal failure (Blood Urea Nitrogen 55 mg/dL, creatinine 4.8 mg/dL), which were new compared to two weeks prior. Her hemoglobin, liver enzymes, coagulation panel, and lactate dehydrogenase (LDH) were within normal limits. Urinalysis revealed pyuria, microscopic hematuria, mild proteinuria, and culture grew* Escherichia coli*. She was treated with intravenous fluids, zoledronic acid, and antibiotics, and her renal function and electrolytes improved. A Computed Tomography (CT) scan of the chest/abdomen/pelvis with oral contrast (Figure 1) revealed a thickened peritoneum and omentum (arrowheads) and ascites (arrows), consistent with peritoneal carcinomatosis. Visualization of her pelvis was limited due to streak artifact from bilateral hip prostheses. She had no evidence of obstruction, mass lesions, or lymphadenopathy, and her visualized organs were normal in appearance. 

Her past medical history was remarkable for hypertension, diabetes mellitus, osteoarthritis, and coronary artery disease. She denied any use, past or present, of alcohol, tobacco, or illicit drugs. She was of Jewish descent and lived in the US her entire life as a homemaker, with no history of foreign travel. She lived alone and was very functional at baseline. Menses began at age thirteen, and menopause at age fifty. She had two children who were alive and well. She was up to date with her health maintenance and cancer screening, and she had no history of abnormal pap smear, mammogram, or colonoscopy. She had no family history of malignancy.

Serum tumor markers CEA and CA 19-9 were normal, CA-125 was mildly elevated at 94 U/mL (normal range: 0–37 U/mL). Endovaginal ultrasound was performed showing normal appearing ovaries. Diagnostic paracentesis was performed, which showed a serum-ascites albumin gradient (SAAG) < 1.1, with negative culture. Abdominal fluid cytology showed squamoid-appearing cells suspicious for, but nondiagnostic of, malignancy. 

Malignancy remained the most likely diagnosis, and a tissue diagnosis was indicated for further management; however, prior to undergoing an open biopsy and possible debulking, she suffered a non-ST-elevation myocardial infarction (MI). During recovery from the MI, she was noted to have feculent material in her urinary catheter bag. A CT scan confirmed the presence of an enterovesicular fistula. She underwent urgent exploratory laporotomy, which revealed a large dense tumor mass involving the midline periumbiical area contiguous with the bladder, intraperitoneal enteric contents, and a perforation was identified in the dome of the bladder. The bladder dome was excised and closed in the three layers. Bladder tumor and peritoneal biopsies were obtained for pathologic assessment.

**Figure 2:**
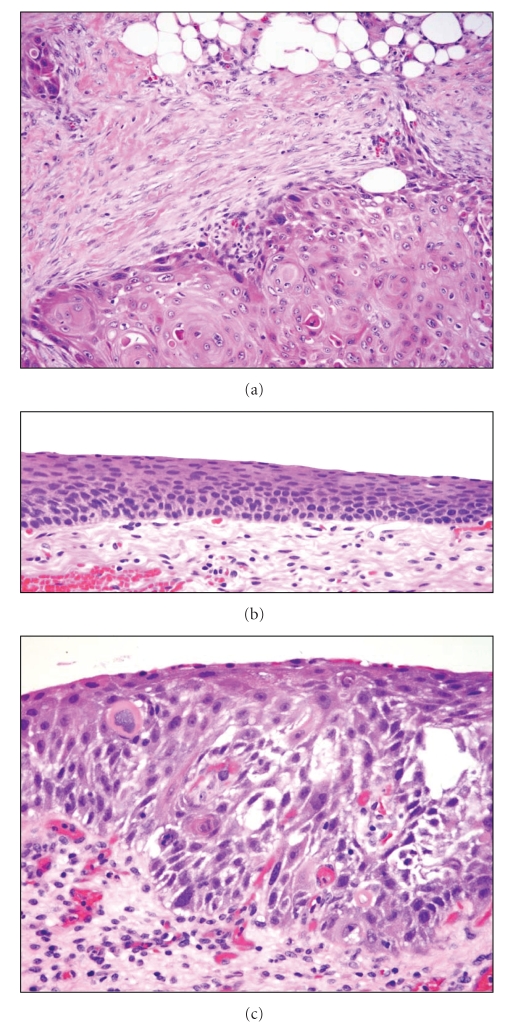
Panel (a) shows an area of invasive, keratinizing squamous cell carcinoma that has infiltrated beyond the muscularis propria (detrusor muscle) and into the perivesical adipose tissue. There is an associated desmoplastic reaction to the tumor. Panel (b) demonstrates the extensive squamous metaplasia seen in the background benign urothelium. Adjacent to the invasive tumor were foci of squamous cell carcinoma *in situ *(Panel (c)). Original magnification—200× (panel (a)), 400× (panels (b), and (c)).

The peritoneal biopsy showed invasive squamous cell carcinoma, keratinizing type. The bladder also showed invasive keratinizing squamous cell carcinoma involving the full thickness of the wall and extending into the perivesical soft tissue. The adjacent bladder mucosa showed focal squamous cell carcinoma *in situ* and extensive squamous metaplasia of the surrounding benign urothelium (Figure 2). Panel (a) shows an area of invasive, keratinizing squamous cell carcinoma that has infiltrated beyond the muscularis propria (detrusor muscle) and into the perivesical adipose tissue. There is an associated desmoplastic reaction to the tumor. Panel (b) demonstrates the extensive squamous metaplasia seen in the background benign urothelium. Adjacent to the invasive tumor were foci of squamous cell carcinoma *in situ *(Panel (c)). The combination of these findings supported the final diagnosis of primary squamous cell carcinoma of the urinary bladder.

Shortly after the fistula repair, she began to clinically decline with a deterioration of her mental status, worsening pain, and concern for progressive disease. The decision was made by her family not to pursue aggressive treatment. She died on hospital Day 23 and autopsy was declined.

## 2. Discussion

Peritoneal carcinomatosis is a common presentation of selected advanced stage tumors, or as a later complication of disease progression or recurrence in numerous tumor types. In adult females, the most common etiology is ovarian adenocarcinoma, although carcinomas of the gastrointestinal tract, lung, or breast, or primary peritoneal carcinoma can produce this clinical syndrome. Other rarer tumor types that should be considered are cervical, endometrial, peritoneal mesothelioma, or lymphoma. In addition to looking for the primary tumor site, ascitic fluid cytology may be useful in making the diagnosis. However, the sensitivity of cytology ranges from 58–75% [1], depending on the number of specimens processed, the quality of the specimens, and the tumor type. Serum tumor marker elevation is common, but is nonspecific and can be misleading [2]. Omental biopsy is often performed in patients to verify malignancy when a primary site cannot be established. Women with normal appearing ovaries, if no other apparent primary site is found, are usually considered to have primary peritoneal carcinoma [3].

Squamous cell carcinoma (SCC) of the bladder is a rare malignancy in the United States, accounting for 1–3% of bladder tumors [4]. It is most common in the 7th decade of life, and males and African Americans are most likely to be affected. Infection with the parasite *Schistosoma haematobium* is an important risk factor in parts of the world where the organism is endemic. However, in the United States nearly all cases are nonschistomal in origin. Both schistosomal and nonschistosomal cases of SCC of the bladder typically show a background of squamous metaplasia, and in many cases SCC *in situ *may be present, which provides indirect evidence that the invasive SCC is in fact a primary bladder squamous carcinoma [5]. In the United States, risk factors for SCC of the bladder include those situations that commonly induce keratinizing squamous metaplasia, a process that results from repeated urothelial injury. Thus, recurrent urinary tract infections, bladder calculi, radiation exposure, chronic indwelling catheters, neurogenic bladder, and cigarette smoking are all risk factors. Interestingly, our patient lacked exposure to all of these established risk factors.

Nearly all patients with SCC of the bladder present with hematuria. Our patient did not have gross hematuria, and microscopic hematuria was initially attributed to her underlying urinary tract infection. Less common presenting symptoms can include irritative bladder symptoms, weight loss, and urinary obstruction. The majority of bladder SCCs are high grade, high stage tumors with most cancers having muscle invasion at the time of diagnosis. If on biopsy a diagnosis of squamous cell carcinoma *in situ *is made without an invasive component identified, the clinician should consider rebiopsy or close clinical follow up, as most patients with SCC *in situ *either concurrently have muscle-invasive SCC or will develop muscle-invasive disease within months of the diagnosis of *in situ *SCC. Of note, in order to label a bladder tumor as a pure squamous cell carcinoma, thorough pathologic sampling should be performed to exclude the presence of an invasive high grade urothelial carcinoma component. If the latter is, in fact, documented, the tumor should not be referred to as a squamous cell carcinoma but rather termed (and treated as) invasive high grade urothelial carcinoma with squamous differentiation. The finding of squamous metaplasia, as well as SCC *in situ*, as in our case, is also helpful in determining that a tumor is a true primary squamous cell carcinoma of the bladder as opposed to urothelial carcinoma with squamous differentiation or metastasis from another site. 

There are limited data available regarding how to treat patients with advanced SCC of the bladder as they tend to be excluded from clinical trials studying urothelial carcinoma. Treatment of clinically localized disease is usually surgical since these tumors may be resistant to chemotherapy and radiation, similar to squamous cell carcinomas of other sites [6]. Chemotherapeutic regimens for urothelial carcinoma are sometimes used but with variable results [7, 8], though chemotherapy for nonbilharzial SCC is under-studied. Prognosis for patients with SCC of the bladder is poor, and most die from their disease within 1–3 years of diagnosis. The reported 5-year survival rate is 33–48% [9]. Death is usually due to locoregional progression (oftentimes ureteral or bladder neck obstruction and subsequent renal failure), and distant metastases are rare.

Primary squamous cell carcinoma of the bladder presenting as peritoneal carcinomatosis has not been reported in the literature. In patients who present with a clinical picture of peritoneal carcinomatosis, primary bladder SCC, as well as carcinomas of the urinary tract in general, could be considered in the differential diagnosis, particularly when aggressive and potentially morbid procedures are being considered (e.g., surgical debulking for presumed primary peritoneal carcinomatosis).
